# Editorial: Women in plant physiology: 2022

**DOI:** 10.3389/fpls.2023.1203958

**Published:** 2023-05-26

**Authors:** Alessandra Boccaccini, Lydia Pui Ying Lam

**Affiliations:** ^1^ Center for TeleInfrastructure (CTIF), University of Rome Tor Vergata, Rome, Italy; ^2^ Graduate School of Engineering Science, Akita University, Akita, Japan

**Keywords:** plant physiology, environment, photosynthesis, abiotic stress, biotic stress, biotechnology

Plants are essential for life on earth, not only because they produce oxygen *via* photosynthesis, but also because our everyday life highly depends on them. They provide us with clothes, food, and shelter. Ancient and modern medicine use plant metabolites as drugs for disease treatment. Moreover, plants can be engineered to produce vaccines, antibodies, and pharmaceutical proteins. Plants have a positive impact on the ecosystem, cleaning the environment by filtering toxic contaminates from the air, soil, and water. All these aspects demonstrate the importance of studying plants because only a deep understanding of how plants work can ensure smart exploitation of them and the development of new solutions for pressing problems. Hence, this Research Topic wanted to embrace all aspects of plant physiology, giving a voice to less-represented scientists in this field. According to UNESCO, women represent only 30% of the world’s researchers. Hence, by promoting their works through this dedicated Research Topic on Women in Plant Physiology, Frontiers wanted to support women’s careers, reducing the gender gap.

Half of the papers in this collection focused on studying plant-environment interactions (**Figure 1**). Since plants are sessile organisms that cannot flee or change their habitats depending on the seasons and nutrient availability, they have evolved mechanisms to cope with the ever-changing environment by adapting their growth and development to it. Among the external stimuli, light is one of the most important because it is required in photosynthesis to provide energy. Dukic et al. used *Arabidopsis thaliana* to dissect the molecular mechanism that regulates photosynthesis in the presence of limiting light. The authors found that phosphorylation of the light-harvesting complex II, happening in low light, depends on the Cl^−^ channel ClCe that regulates H^+^ flux and ATP synthase activities. Moreover, phenotypic analyses of the *clc* mutant demonstrates the importance of this channel in controlling the acclimation of plants under low light. In addition, Mahati and Padmasree studied photosynthesis, focusing on the effect of brassinolide on the crosstalk between mitochondria and chloroplasts to regulate the Calvin-Benson cycle.

**Figure 1:**
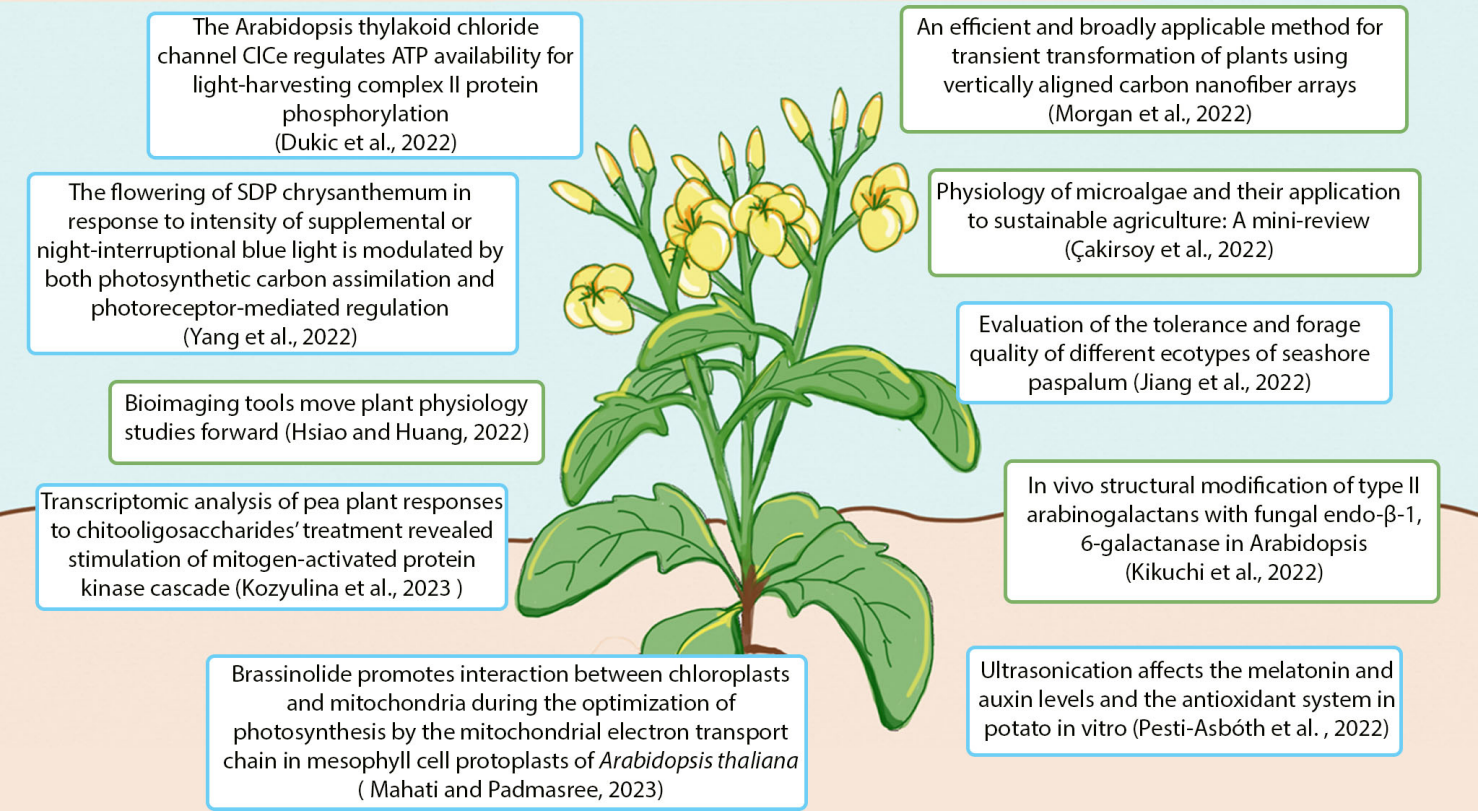
Summary of the contents of the Research Topic “Women in Plant Physiology.” Blue boxes represent articles focused on plant-environment interactions, while green boxes indicate articles that discussed biotechnology and applied science.

Light is not only a source of energy, but also provides information about the environment. The number of hours of light during the day is used by plants to determine the season, which is critical to decide when to flower (Freytes et al., 2021). The flowering time is particularly important in commercial plant species. For this reason, Yang et al. studied how supplementing with blue light during the day or an interrupting blue light pulse during the night affects the flowering of the Gaya Glory cultivar of *Chrysanthemum morifolium*.Even if plants are great at responding to environmental changes, some of these changes are considered stresses that negatively affect plant fitness. However, some species have evolved to grow in habitats that are hostile to other species. This is the case for *Seashore paspalum*, which can grow in the presence of a high concentration of salt, as presented by Jiang et al. These authors analyzed the salt tolerance and forage quality of 16 different ecotypes of *Seashore paspalum*, suggesting that the ecotype with the best salt-resistant phenotype and high nutritional quality could be exploited in agriculture and animal husbandry. Finding salt-resistant genotypes is particularly important for modern agriculture, which is endangered by soil salinization aggravated by climate change and anthropogenic activities (Mukhopadhyay et al., 2021).

Ultrasound is an abiotic stress that impacts plant growth and development. Pesti-Asbóth et al. discovered that ultrasonication reduces the growth and chlorophyll content of potato, activates antioxidant pathway, increases auxins production, and decreases melatonin accumulation. Their data provide new insights into how plants cope with ultrasound stress.

In addition to abiotic stresses, plants are also often attacked by herbivores and pathogens such as fungi, viruses, and bacteria. The mechanisms used to respond to pathogen attack are known as plant immunity, and can be elicited by natural compounds such as chitooligosaccharides. The article by Kozyulina et al. describes the transcriptional response to chitooligosaccharides of the pea plant, identifying several cellular pathways affected by this treatment and demonstrating the importance of studying plant elicitors in crops.

The other half of this collection focused on applied science and biotechnology (**Figure 1**). For sustainable agricultural development, microalgae have emerged as a renewable alternative to chemical fertilizers. Microalgae could efficiently take up nutrients from wastewater, and their biomass could be used as fertilizers. Çakirsoy et al. described and highlighted the potential of using microalgae for nutrient recycling, their physiological properties that enable rapid growth and efficient nutrient uptake, the properties and advantages of microalga-based fertilizers, as well as challenges in their actual applications.

Arabinogalactan-proteins (AGPs) are plant extracellular glycoproteins that are decorated by type II arabinogalactans (Ma and Johnson, 2023). To study the functions of the long β-1,6-galactohexaose (β-1,6-Gal6) side chains of AGPs, Kikuchi et al. generated transgenic arabidopsis with reduced long β-1,6-galactan side chains by expressing a dexamethasone inducible fungal endo-β-1,6-galactanase, Tv6GAL, that could degrade these long side chains. After dexamethasone treatment, these plants showed retarded growth, higher extensibility, lower breaking load, and reduced cellulose content, suggesting the role of long β-1,6-galactan side chains in cellulose production and/or deposition, which could influence cell growth.

Finally, tools for studying plant physiology have been developed or summarized. Transient gene expression in plants is widely employed to study gene functions. Morgan et al. developed an efficient transient transformation method that could be applied to different plant species (Arabidopsis, poplar, lettuce, *Nicotiana benthamiana*, and tomato) and organs by using vertically aligned carbon nanofiber arrays. This method holds potential for application in species that are recalcitrant to being transformed by *Agrobacterium*. Bioimagining tools are also widely used in the study of plant physiology. Their applications span from tracking morphogenesis to visualizing macromolecular dynamics. Hsiao and Huang summarized the key contributions of female scientists to the development of bioimaging techniques, which have moved plant physiology study forward. In addition, this opinion article also describes the history of these female scientists, aiming to encourage and inspire the next generation of female researchers to grow their interest in studying plant physiology and cell biology.

Hence, this Research Topic addresses a variety of questions in the plant physiology field and highlights the necessity of constant crosstalk between basic and applied research to move forward the knowledge in this field.

## Author contributions

All authors listed have made a substantial, direct, and intellectual contribution to the work and approved it for publication.
